# Supplementary material for: Endogenic Phenolic Compounds of Barley as Potential Biomarkers Related to Grain Mycotoxin Production and Cultivar Selection
Source: Biology (Basel). 2023 Oct 3;12(10):1306. doi: 10.3390/biology12101306 (PMC10604134; doi:10.3390/biology12101306)
Supplement: Supplementary file 1 [file biology-12-01306-s001.zip › biology-2607566-supplementary.pdf]

Supplementary materials

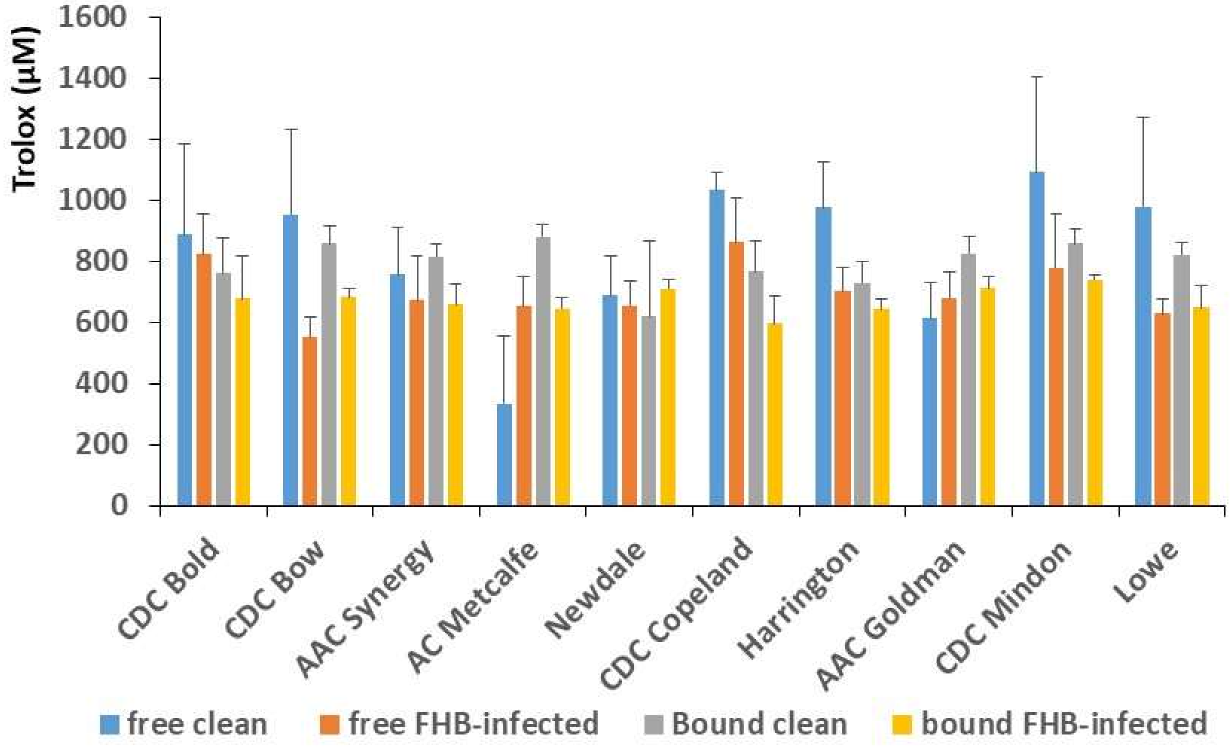

Figure S1. Antioxidant activity assay on free and bound fractions of clean and FHB-infected barley.

A.

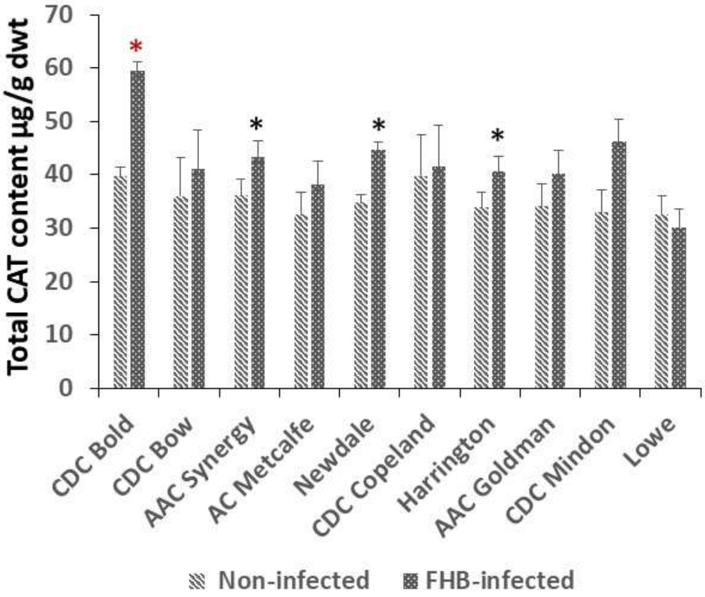

B

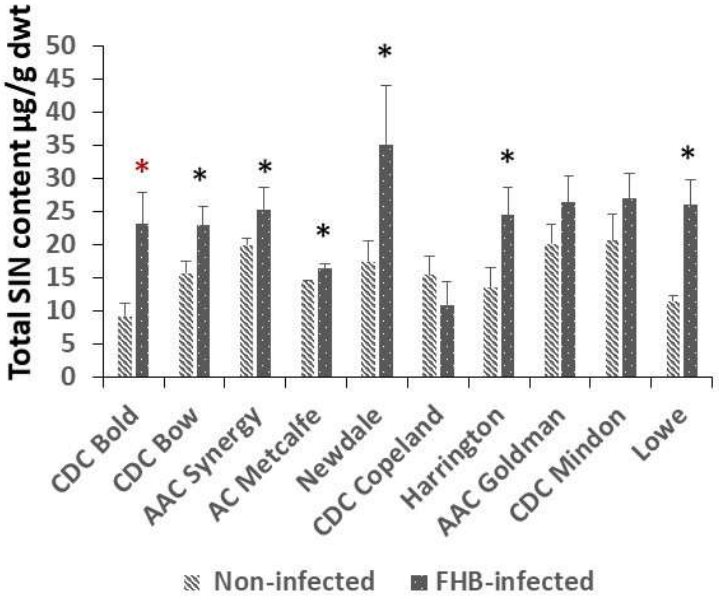

Figure S2. Changes in total (both free and bound) catechin (A) acid and sinapic acid (B) contents before and after FHB infection. *Significant at level p≤0.05 compared to the contents in non-infected (clean) grains of the tested cultivars. Susceptible check significant change is shown with * .

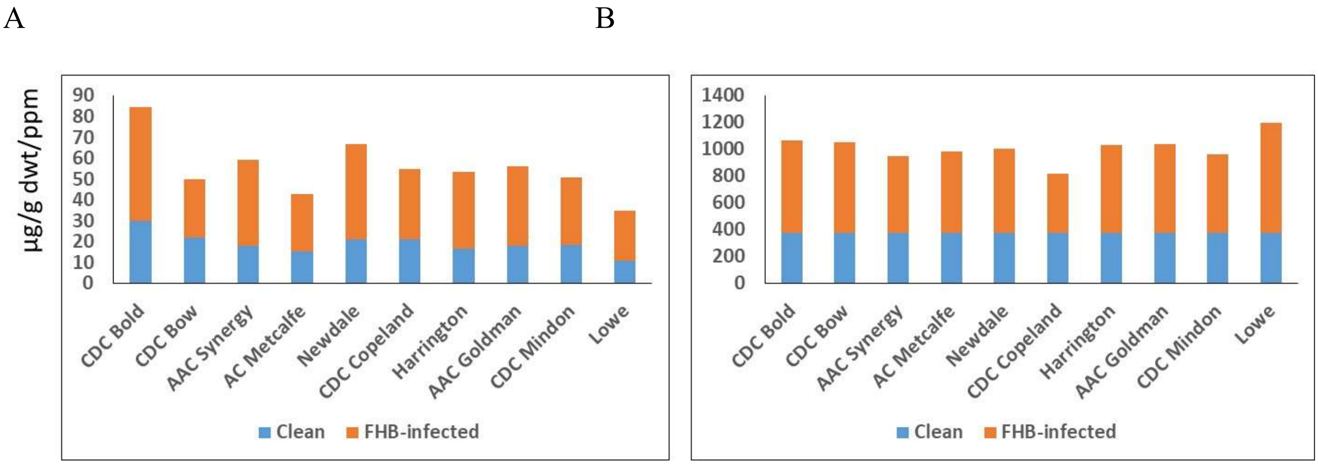

Figure S3. The sum of total individual phenolic compound contents of free (A) and bound (B) barley phenolic extracts. Blue shows clean extracts and orange shows FHB-infected barley grain extracts.

A)

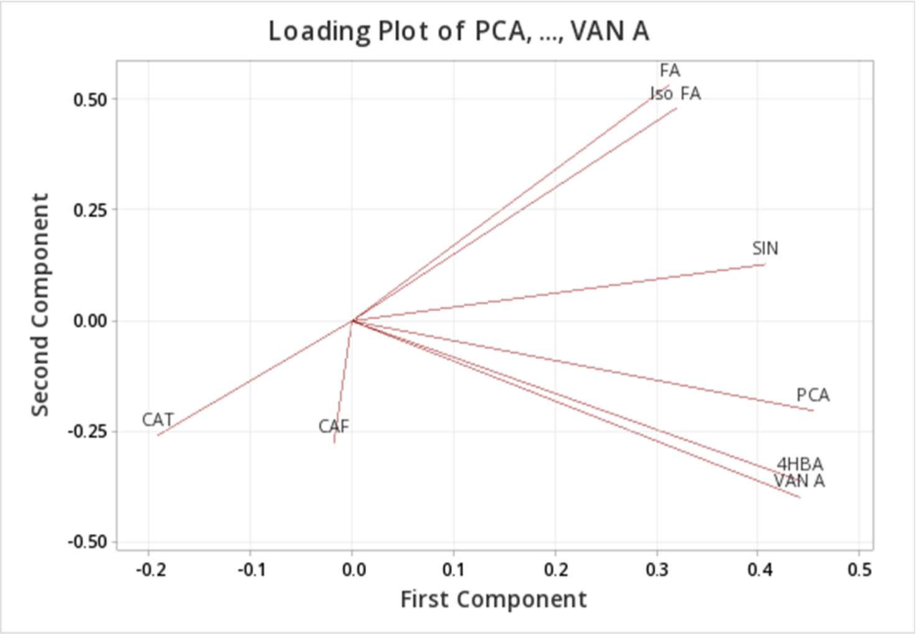

B)

Variable	PC1	PC2	PC3	PC4	PC5
**PCA**	0.455	-0.202	-0.184	-0.065	0.625
**CAF**	-0.018	-0.275	0.598	0.630	0.064
**SIN**	0.407	0.128	-0.467	0.290	-0.439
**FA**	0.313	0.532	0.033	0.031	0.400
**Iso FA**	0.320	0.479	0.242	0.373	-0.129
**CAT**	-0.192	-0.259	-0.533	0.592	0.243
**4HBA**	0.442	-0.360	0.000	-0.077	-0.410
**VAN A**	0.441	-0.397	0.214	-0.133	0.091

Figure S4. The principal component and cluster analysis of the selected phenolic compounds in tested 10 barley cultivars; (A) Loading plots of the principal component analysis; blue dots indicate each barley cultivar tested; (B) Eigenvectors for each principal component.

Table S1. The background information of cultivars used for the study

Name	Class	Year of Registration	FHB reaction	Origin/Breeding program*	Reference
**1. CDC Bold**	General purpose	1999	S (check)	Canada/ U of S-CDC	CFIA (Canadian Food Inspection Agency)
**2. CDC Bow**	Malting	2016	MS	Canada/ U of S-CDC	CFIA
**3.AAC Synergy**	Malting	2012	I-MS	Canada/ AAFC-BRDC	Legge et al., 2014
**4.AC Metcalfe**	Malting	1997	I	Canada/ AAFC-BRDC	Legge et al., 2003
**5. Newdale**	Malting	2007	I	Canada/ AAFC-BRDC	Legge et al., 2008
**6.CDC Copeland**	Malting	1999	I	Canada/ U of S-CDC	CFIA
**7. Harrington**	Malting	1981	MR	Canada/ U of S-CDC	Harvey and Rossnagel, 1984
**8.AAC Goldman**	Malting	2018	MR	Canada/ AAFC-BRDC	Legge et al., 2018
**9.CDC Mindon**	General purpose	2007	MR (check)	Canada/ U of S-CDC	Rossnagel et al, 2008
**10. Lowe**	Malting	2016	MR	Canada/ AAF-FCDC	Juskiw et al, 2019

* AAF-FCDC = Alberta Agriculture and Forestry - Field Crop Development Centre, Lacombe, AB; AAFC-BRDC = Agriculture and Agri-Food Canada - Brandon Research and Development Centre, Brandon, MB; and U of S - CDC = University of Saskatchewan - Crop Development Centre, Saskatoon, SK.

Table S2. Free phenolic compounds detected by high-performance liquid chromatography (HPLC) in non-infected (clean) barley cultivars.

Cultivar	Phenolic compound (µg/g dwt)			
	**PCA**	**SIN**	**FA**	**CAT**
CDC Bold	0.109±0.04	0.36±0.1	2.50±0.2	26.9±0.9**
CDC Bow	ND	0.11±0.04	1.79±0.2	20.1±5.1
AAC Synergy	ND	0.23±0.01	2.19±0.1	15.8±1.1
AC Metcalfe	ND	0.17±0.08	1.79±0.1	13.3±1.0
Newdale	0.067±0.03	0.15±0.01	2.09±0.1	18.7±0.8
CDC Copeland	0.056±0.02	0.34±0.2	2.48±0.2	18.3±0.6
Harrington	ND	0.35±0.04	2.78±0.1	13.8±1.0
AAC Goldman	ND	0.19±0.1	2.00±0.1	16.1±1.4
CDC Mindon	ND	0.42±0.2	2.61±0.1	15.5±1.0
Lowe	0.283±0.03	0.25± 0.1	2.44±0.1	8.1±0.5*

ND- Not detected

PCA- P coumeric acid, SIN- sinapic acid, FA- ferulic acid, CAT- catechin

**- Highest content of the specific phenolic compound

*-Lowest content of the specific phenolic compound

Table S3. Free phenolic compounds detected by HPLC in FHB-infected barley cultivars.

Cultivar	Phenolic compound (µg/g dwt)			
	**SIN**	**FA**	**Iso FA**	**CAT**
CDC Bold	10.8±3.6**	2.2±0.1	0.36±0.1	41.2±5.7**
CDC Bow	1.52±0.8*	1.6±0.1	ND	24.9±4.2
AAC Synergy	7.58±2.8	2.2±0.3	1.96±0.5	29.5±3.5
AC Metcalfe	2.27±0.1*	1.8±0.3	0.21±0.1	23.2±0.7
Newdale	12.8±6.3**	2.3±0.2	1.11±0.4	29.6±2.1
CDC Copeland	4.76±2.0	1.9±0.4	0.63±0.2	26.3±3.7
Harrington	7.71±2.1	2.3±0.6	0.38±0.2	26.4±4.2
AAC Goldman	7.15±2.8	2.1±0.2	1.63±0.2	26.9±4.7
CDC Mindon	7.69±1.1	1.9±0.4	0.92±0.2	21.7±5.2
Lowe	7.66±1.6	2.4±0.2	0.62±0.2	13.2±2.8*

ND- Not detected
SIN- sinapic acid, FA- ferulic acid, Iso-FA- Iso ferulic acid, CAT- catechin
**- Highest content of the specific phenolic compound
*-Lowest content of the specific phenolic compound

Table S4. Bound phenolic compound contents in different cultivars of non-infected (clean) barley.

**Cultivar**	**Phenolic compound (µg/g dwt)**							
	**PCA**	**CAF**	**SIN**	**FA**	**Iso FA**	**CAT**	**4HBA**	**VAN A**
CDC Bold	140±17*	8.6±2.6 *	8.9±1.9*	191±46.5*	12.2±4.1*	12.8±0.8*	1.81±0.27	4.85±0.25*
CDC Bow	228±53	9.9±0.7	15.7±1.7	293±41.5	14.2±1.8	15.9±2.1	1.88±0.07	6.74±1.23
AAC Synergy	214±22	12.8±1.0	19.8±0.9	382±14.3	22.5±1.0	20.3±2.0	2.90±0.32	9.21±1.21
AC Metcalfe	202±23	11.1±0.8	14.4±0.1	338±15.4	20.9±1.3	19.2±3.3	2.81±0.09	9.88±1.71**
Newdale	262±45	11.7±1.4	17.4±3.0	359±47.7	18.9±3.5	16.1±3.7	2.81±1.01	9.25±0.34
CDC Copeland	236±34	10.6±2.1	15.2±2.5	342±4.2	16.9±2.9	21.5±7.1	1.64±0.24*	8.19±1.49
Harrington	209±27	13.5±2.1	13.3±2.9	469±101**	24.2±7.0**	20.2±1.8	1.88±0.13	8.41±0.08
AAC Goldman	245±54	14.5±0.8	20.0±2.9**	377±33.2	21.8±1.4	18.0±2.9	3.18±0.12**	9.33±1.41
CDC Mindon	265±35	15.8±3.3**	20.3±3.7**	375±51.5	22.5±5.5	17.5±3.0	2.33±0.1	9.37±1.65**
Lowe	331±96**	10.4±1.1	11.3±0.8	368±25.5	17.4±1.5	24.6±2.9**	2.21±0.25	9.41±1.45

Table S5. Bound phenolic compound contents in different cultivars of FHB-infected barley.

Cultivar	Phenolic compound (µg/g dwt)							
	PCA	CAF	SIN	FA	Iso FA	CAT	4HBA	VAN A
CDC Bold	305±38	11.0±1.3	12.5±1.1*	317±54.3	14.6±2.7	18.3±3.3	2.63±0.36	9.12±1.70
CDC Bow	308±80	8.7±1.4	21.4±2.1	293±59.5	13.9±1.4*	16.2±1.6	2.34±0.42	8.82±0.42
AAC Synergy	206±22	10.6±1.6	17.8±0.6	293±72.0	16.6±2.7	13.8±1.3	2.35±0.36	8.93±0.91
AC Metcalfe	238±9	11.3±0.5	14.2±0.6	300±51.7	15.2±0.7	15.0±1.7	2.35±0.13	9.69±1.29
Newdale	261±42	9.2±0.5	22.4±2.6**	288±48.8	14.2±3.2	15.1±1.9	3.68±0.77**	9.65±0.20
CDC Copeland	152±31*	10.5±1.2	10.2±1.5	228±28.0*	11.4±1.7*	15.3±0.4	2.51±0.71	8.21±2.51
Harrington	177±43	8.4±2.2*	16.8±2.2	412±84.6	19.0±8.9**	14.3±2.3	1.61±0.13*	7.20±1.62*
AAC Goldman	263±21	10.3±1.2	19.3±1.3	322±16.4	18.0±3.3	13.4±0.9*	3.07±0.32	9.51±0.98
CDC Mindon	208±50	12.2±1.9**	19.4±2.6	304±53.5	18.3±4.2	14.6±1.0	2.41±0.36	8.12±1.98
Lowe	325±67**	10.6±1.0	18.4±2.2	417±73.5**	19.9±4.7**	16.9±0.6**	3.43±1.18	10.1±0.91**
